# Immuno-Interface Score to Predict Outcome in Colorectal Cancer Independent of Microsatellite Instability Status

**DOI:** 10.3390/cancers12102902

**Published:** 2020-10-09

**Authors:** Ausrine Nestarenkaite, Wakkas Fadhil, Allan Rasmusson, Susanti Susanti, Efthymios Hadjimichael, Aida Laurinaviciene, Mohammad Ilyas, Arvydas Laurinavicius

**Affiliations:** 1 National Center of Pathology, Affiliate of Vilnius University Hospital Santaros Klinikos, 08406 Vilnius, Lithuania; allan.rasmusson@vpc.lt (A.R.); aida.laurinaviciene@vpc.lt (A.L.); arvydas.laurinavicius@vpc.lt (A.L.); 2 Institute of Biosciences, Life Sciences Center, Vilnius University, 10257 Vilnius, Lithuania; 3 Molecular Pathology Group, Unit of Academic Molecular Pathology, Division of Cancer and Stem Cell, School of Medicine, Queen’s Medical Centre, University of Nottingham, Nottingham NG7 2UH, UK; wakkas.fadhil@nottingham.ac.uk (W.F.); susanti.susanti@nottingham.ac.uk (S.S.); efthymios.hadjimichael2@nuh.nhs.uk (E.H.); mohammad.ilyas@nottingham.ac.uk (M.I.); 4 Nottingham Molecular Pathology Node, Queen’s Medical Centre, University of Nottingham, Nottingham NG7 2UH, UK; 5 Department of Pathology, Forensic Medicine and Pharmacology, Faculty of Medicine, Institute of Biomedical Sciences, Vilnius University, 03101 Vilnius, Lithuania

**Keywords:** tumor infiltrating lymphocytes, tumor microenvironment, Immunogradient, CD8, CD20, tumor growth pattern, immuno-interface score, colorectal cancer

## Abstract

**Simple Summary:**

For pathologists, how to precisely diagnose cancer from microscopy slides of tumor tissue samples so that each patient may receive the optimal treatment for his specific type of disease is a major task. Recent research based on digital pathology image analysis enables new approaches to assess tumor-host interaction at a microscopic level. The current study applies a novel spatial analysis method which computes Immunogradient indicators to estimate the migration of immune cells towards the tumor across the tumor/stroma interface. These indicators, computed for two types of immune cells (CD8 and CD20), proved to be independent prognostic factors in this study of 87 patients with colorectal cancer. The indicators were combined with infiltrative tumor growth pattern, assessed by a pathologist, into a new immuno-interface score which enabled prediction of the patient survival independent of other clinical, pathology and molecular characteristics of the tumor. The study demonstrates the value of computational pathology to advance the precision of clinical decision-making.

**Abstract:**

Tumor-associated immune cells have been shown to predict patient outcome in colorectal (CRC) and other cancers. Spatial digital image analysis-based cell quantification increases the informative power delivered by tumor microenvironment features and leads to new prognostic scoring systems. In this study we evaluated the intratumoral density of immunohistochemically stained CD8, CD20 and CD68 cells in 87 cases of CRC (48 were microsatellite stable, MSS, and 39 had microsatellite instability, MSI) in both the intratumoral tumor tissue and within the tumor-stroma interface zone (IZ) which was extracted by a previously developed unbiased hexagonal grid analytics method. Indicators of immune-cell gradients across the extracted IZ were computed and explored along with absolute cell densities, clinicopathological and molecular data, including gene mutation (*BRAF*, *KRAS*, *PIK3CA*) and MSI status. Multiple regression modeling identified (*p* < 0.0001) three independent prognostic factors: CD8+ and CD20+ Immunogradient indicators, that reflect cell migration towards the tumor, were associated with improved patient survival, while the infiltrative tumor growth pattern was linked to worse patient outcome. These features were combined into CD8-CD20 Immunogradient and immuno-interface scores which outperformed both tumor-node-metastasis (TNM) staging and molecular characteristics, and importantly, revealed high prognostic value both in MSS and MSI CRCs.

## 1. Introduction

Colorectal cancer (CRC) is globally the third most commonly diagnosed and second leading cause of cancer-related deaths for both sexes [1]. Recent improvements in survival are associated with both earlier disease detection and the development of personalized tumor biology-based therapies [1,2]. The main factor in cancer management however is still the traditional tumor-node-metastasis (TNM) staging system. Although this provides very powerful and robust prognostic information, there is wide variation in the outcome of patients within individual stage categories [3]. The precision in identifying the patients at high risk of tumor progression and those who may benefit from combined therapies could be improved by including the information on the molecular profiles of tumors and “immune” community in the tumor microenvironment (TME) to the TNM system [4,5,6].

Currently, only a few molecular markers have been implemented for the management of CRC although these have mainly been for therapy stratification such as testing for activating *KRAS*, *NRAS* and *BRAF* gene mutations as exclusion criteria for the use of EGFR-targeted therapies in metastatic CRC (mCRC) [2]. Although *RAS* and *BRAF* mutations are considered to be poor prognostic factors [7], outside of targeted therapies, they are not used for outcome predictions in routine CRC diagnostics. Recently, tumor microsatellite instability (MSI) traditionally used to identify Lynch syndrome patients [8], was rediscovered as a biomarker for immunotherapy in CRC [9,10]. Tumors with MSI are highly immunogenic due to loss of DNA mismatch repair function. This results in an increased mutation rate with consequent generation of neo-antigens stimulating an anti-tumor immune response which is considered as the basis of improved patient survival [11,12]. CRC MSI tumors have been shown to be enriched with checkpoint proteins like PD-1, PD-L1, and CTLA-4 that are targeted clinically with immune checkpoint inhibitors [13]. Over the last decade, comprehensive research of the TME, especially the cancer immunome and local cell infiltrates, has led to the recognition of host immunity as one of the major factors in cancer biology [14,15]. Tumor infiltrating lymphocytes (TIL) can be viewed as a surrogate marker of the anti-tumor immune response and, histologically, tumors can be seen to be “immune hot” (containing large numbers of TIL) and “immune cold” (containing few TIL) [16]. A number of studies, using both visual assessment and digital image analysis (DIA), have demonstrated that TIL are highly prognostic markers associated with better patient survival in various malignancies including lung, breast, melanoma, pancreas and CRC [17,18,19,20,21].

Digital immunohistochemistry (IHC) based methods have been demonstrated to increase the informative power of immune cell quantification in cancers [22,23]. The Immunoscore^®^ method based on direct quantification of CD3+ and CD8+ cell densities in the core of tumor (CT) and its invasive margin (IM) first proposed in 2012 [24], was in 2018 shown to be a prognostic score superior to TNM-staging in CRC [25,26]. Moreover, Immunoscore^®^ and other immune assessment approaches have shown TIL to be a stronger predictor of tumor recurrence than microsatellite instability status [27,28,29]. Furthermore, immune response assessment and Immunoscore^®^, as a reference method that has demonstrated the immune component to be of the highest relative contribution to the tumor recurrence risk compared to all clinical parameters in CRC [6], was proposed to complement the World Health Organization classification of tumors of digestive system [30]. 

DIA-based immune profiling analysis was further advanced by Nearchou et al. by a combined tumor budding and immune cell quantification and proximity analysis; they proposed a spatial immuno-oncology index based on spatial relationships between tumor buds, TIL and macrophage populations within TME which enabled significant prognostic stratification of stage II CRC [31,32]. Recently, Rasmusson et al. proposed a set of Immunogradient indicators which quantifies the immune cell density gradient across the tumor-stroma interface zone (IZ), sampled by an automated hexagonal tiling statistical modeling; the Immunogradient indicators for CD8+ cell density provided independent prognostic value in CRC and hormone receptor positive breast cancer patients [33]. 

In this study we investigated the prognostic value of immune cell density and Immunogradient indicators for CD8+, CD20+ and CD68+ in the context of MSI status and a variety of clinicopathological and molecular features in a selected CRC patient cohort. Based on identified three independent prognostic indicators (CD8+ and CD20+ Immunogradient indicators and the histological feature of infiltrative tumor growth pattern), we computed CD8-CD20 Immunogradient score and immuno-interface score (IIS) for CRC which is a potential MSI status-independent prognostic tool based exclusively on TME features.

## 2. Results

### 2.1. Patient Clinicopathological Characteristics

The study was performed in an 87 CRC patient cohort with formalin-fixed paraffin embedded (FFPE) surgical resection specimens tested for microsatellite and gene mutation status. A comparison of the clinicopathological and tumor molecular features showed that patients with MSI tumors were older, as has been previously observed in sporadic CRC [34,35]; MSI tumors were associated with poor differentiation by histology, *BRAF* mutations and right-sided location, whereas MSS tumors displayed a higher frequency of *KRAS* mutations; this is in line with observations in other studies [36,37]. There were no associations between MSI status and patient age, sex, the TNM-staging, lymphovascular invasion, perineural invasion, tumor growth pattern or budding (Table 1).

### 2.2. Summary Statistics of Immunogradient and Intratumoral Immune Cell Density Indicators

Cell densities were calculated for all biomarkers in both the intratumoral tissue and inside the IZ, which consists of three aspects: tumor (T), tumor edge (TE) and stroma (S); mean CD8+, CD20+ and CD68+ cell densities were calculated within each aspect. The CD8+ and CD68+ cell densities within the aspects of IZ of width 3 (IZ) and the intratumoral densities were higher in MSI than in MSS tumors, whereas no differences in IZ and intratumoral CD20+ cell densities were observed comparing MSI and MSS tumors (Table 2). 

The Immunogradient indicator Center of Mass (CM) for CD8+ or CD68+ cell densities revealed higher cell density gradient towards the T aspect within the IZ of MSI compared to MSS tumors. In contrast, CM for CD20+ cell density indicated more prominent cell density gradient towards the T aspect within the IZ of MSS than MSI tumors (Table 2). Overall, IZ and intratumoral CD8+ and CD68+ cell densities were similar in MSS tumors, whereas a higher CD8+ cell density compared to CD68+ cell density was seen in the aspects of IZ in MSI tumors; IZ and intratumoral CD20+ cell densities were lowest both in MSI and MSS tumors (*p* < 0.05). CD8+ cell density distributions were similar in the S and TE aspects of IZ, the same was seen for CD68+ cell density distributions, however, both cell markers were less abundant in the T aspect of IZ in MSI and MSS tumors (*p* < 0.05). The density of CD20+ cells were highest in the S, less abundant in TE and lowest in T aspect of IZ in MSI and MSS tumors. There were no significant differences between cell densities in the T aspect of IZ and the tumor tissue, both in MSI and MSS tumors.

### 2.3. Associations of Clinicopathological Parameters, Immunogradient and Intratumoral Immune Cell Density Indicators

**Figure 1:**
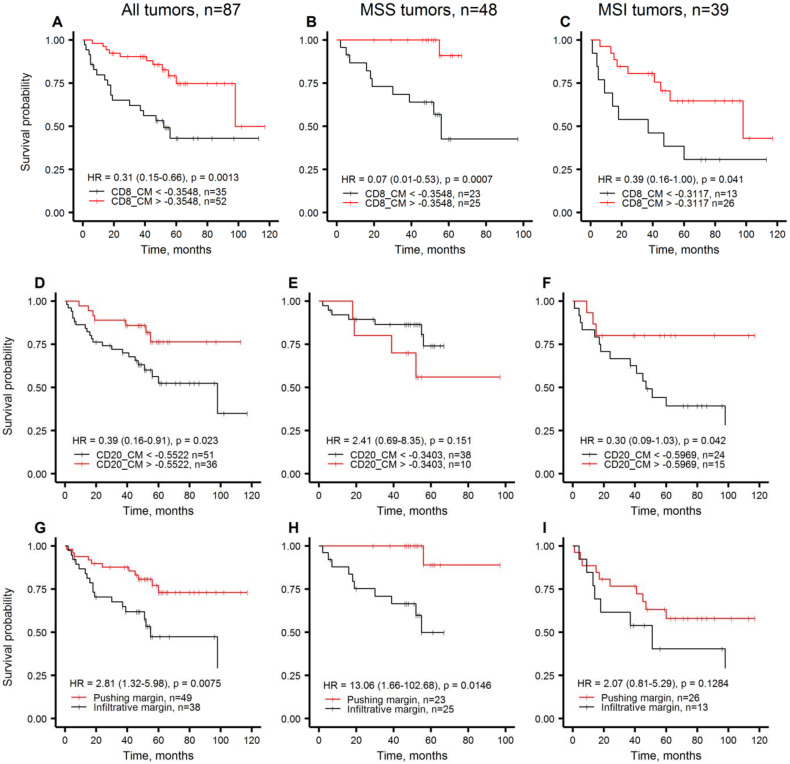
Kaplan-Meier plots representing overall survival probabilities obtained by Immunogradient and histology indicators in all tumors, MSS tumors, MSI tumors, respectively. (**A**–**C**): CM for CD8+ cell density in the IZ; (**D**–**F**): CM for CD20+ cell density in the IZ; (**G**–**I**): tumor growth pattern.

The overall survival (OS) estimates for the clinicopathological parameters and tissue immune response indicators in this cohort of patients are presented in Table 3. For the CM and the cell densities in the IZ aspects, cut-off values were obtained by log-rank test (Cutoff Finder [38]) to stratify the patients into groups with high versus low indicator values. In univariate analysis, statistically significant patient stratifications were obtained by the CM indicator for both CD8+ and CD20+ cell densities, for S and T aspects CD20+ cell densities, and for intratumoral CD20+ cell density, whereas no CD68+ cell density indicators showed any significant associations with the patient outcomes. The OS for Immunogradient-based patient stratifications are presented in Figure 1A,D. CM for CD8+ cell density stratified patients by their 5-year OS probabilities at 75% and 43%; CM for CD20+ cell density provided 5-year OS rates at 76% and 56%. Among the clinicopathological parameters only the tumor growth pattern provided significant prognostic stratification while neither TNM-staging, nor molecular features were associated with the patient OS. The tumor growth pattern predicted 5-year OS at 73% and 47% rates in pushing and infiltrative categories, respectively (Figure 1G). The univariate Immunogradient-based OS stratifications were similar in the subgroups of MSS and MSI tumors (Figure 1 B,C,F), except for CM for CD20+ cell density which did not reach statistical significance (Figure 1E). This finding may indicate a different role of CD20+ cells in MSS tumors; however, additional studies are needed to explore this effect further. The tumor growth pattern revealed significant stratification in MSS and a similar trend in MSI tumors (Figure 1H,I).

**Figure 2:**
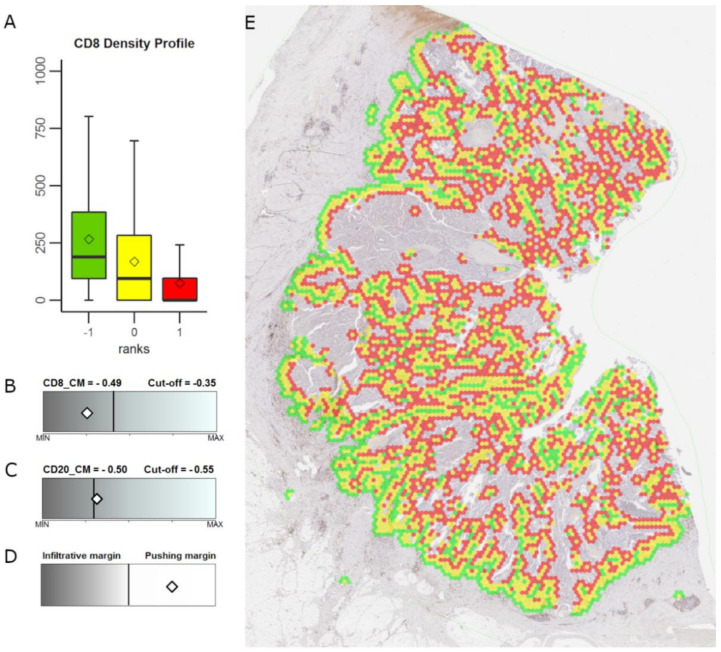
Example of extracted tumor-stroma interface zone, Immunogradient indicator estimates and tumor growth pattern in an individual colorectal cancer case. (**A**): CD8+ cell density profile within the IZ, the box-plot colors correspond to the colors in (**E**); (**B**,**C**): CM for CD8+ and CD20+ cell density values, respectively, represented by a white diamonds within the range of the indicator values; (**D**): tumor growth pattern represented by a white diamond within the two-color bar indicating infiltrative versus pushing margins; (**E**): shows the interface zone overlaid on the whole slide image (see Figure S1 for the original image without the overlay): tumor edge (yellow), tumor aspect (red), stroma aspect (green). Prognostic cut-off values for the CRC patient cohort are represented by the vertical line within the range of the indicator values; the grey shade of the bar represents better prognosis for brighter values and worse prognosis for darker values.

The features that revealed significant patient stratification in univariate analyses (*p* < 0.05, Table 3) were tested for their independent prognostic value by multiple Cox regression (Table 4). A strong prognostic model (Model#1, LR: 23.03; *p* < 0.0001) was obtained with high CM for CD8+ and CD20+ cell densities predicting longer patient OS, and infiltrative tumor growth pattern independently associated with worse patient survival; an example of independent indicators’ estimates in an individual CRC case is presented in Figure 2.

### 2.4. Immuno-Interface Score for Predicting Patient Overall Survival

To integrate the independent informative value of all three indicators, we calculated a combined immune-interface score (IIS) by summing positive prognostic scores obtained from the patient stratifications based on cut-off values for each factor: the CM for both CD8+ and CD20+ cell densities were assigned a value of 1 (favorable) or 0 (unfavorable) for the high and low indicator scores, respectively. Similarly, the tumor growth pattern was assigned a value of 1 (favorable) or 0 (unfavorable) for a pushing or infiltrative tumor margin, respectively. 

**Figure 3:**
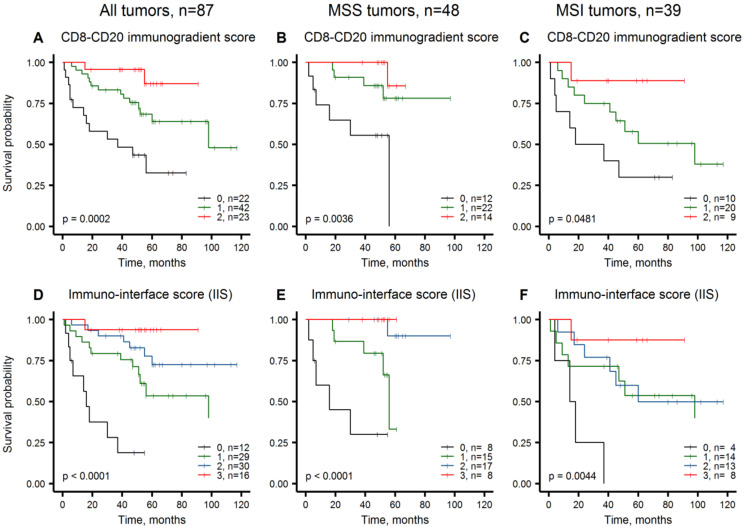
Kaplan-Meier plots representing overall survival probabilities obtained by the combinations of Immunogradient and histology indicators, in all tumors, MSS tumors and MSI tumors, respectively. (**A**–**C**): CD8-CD20 Immunogradient score; (**D**–**F**): immuno-interface score.

Figure 3A outlines patient stratifications obtained by the combined score of CMs for CD8+ and CD20+ cell densities (a combined CD8-CD20 Immunogradient score), which provided three prognostic groups: score 2 with 87%, score 1 with 64%, and score 0 with 33% 5-year OS rates, respectively. Further, we added the prognostic impact of the tumor growth pattern, to calculate IIS, which stratified the patients into four prognostic groups (Figure 3D): score 3 with 94%, score 2 with 73%, score 1 with 53%, score 0 with 19% 5-year OS rates.

## 3. Discussion

This study presents the prognostic value of a novel IIS based on spatial properties of immune response and the tumor-stroma interface histology pattern. Specifically, the score combines the independent prognostic impacts of both CD8+ and CD20+ cell density gradients within the IZ and the tumor growth pattern assessed by pathologist as infiltrative margin. Importantly, the IIS predicted CRC patient OS independently of other clinicopathological and molecular variables, including the MSI status, and provided similar prognostic stratifications in both MSI and MSS subgroups. Furthermore, significant prognostic stratification could be achieved exclusively based on IHC data by computing the combined CD8-CD20 Immunogradient (Model#2, Table 4).

Our study provides further evidence for the value of the recently proposed Immunogradient indicators as independent prognostic factors reported in CRC and hormone receptor-positive breast cancer patients [33]. In particular, the CM for CD8+ cell density within the IZ (CD8+ Immunogradient) was a strong independent predictor of better OS in CRC patients (HR: 0.39, *p* = 0.0071) [33], similar to that in the current analysis of an independent CRC cohort (HR: 0.31, *p* = 0.0029, Table 4). In contrast to other DIA studies, based on enumeration of immune cell densities and their proportions in CT and IM [25] or distances between the cell populations [31,32] that improved prognostic accuracy, the CM indicator represents directional change (gradient) of immune cell density within the stroma-to-tumor transition TME compartment, automatically sampled as IZ. The precision of the sampling and, subsequently, of the Immunogradient computation is therefore less affected by variable tumor growth patterns. Remarkably, the Imunogradient indicators outperform absolute or relative immune cell densities in TME compartments in the multiple prognostic models, both in MSS and MSI tumor subgroups.

In this study, we also tested the prognostic value of CD20+ and CD68+ cell density indicators. Although several studies have shown the prognostic value of tumor-associated macrophage infiltrates in CRC [39,40], we were not able to demonstrate an independent prognostic value of TME CD68+ cell densities. Despite CD20+ cells were less abundant than CD68+ cells and CD8+ cells in tumors, CD20+ cell density features were associated with patient survival. Higher IZ and intratumoral CD20+ cell densities were significantly associated with better patient survival in univariate analyses, except the MSS tumors (Figure 1E, Table 3). Only CD20+ Immunogradient (by CM) was an independent predictor of longer patient OS (Table 4). Previous studies have demonstrated beneficial prognostic impact of B cell infiltrates in TME of various cancers, including primary and metastatic CRCs [41,42,43]. Of note, the CD20+ Immunogradient indicators provided significant patient stratifications in MSI tumors (Figure 1F) but did not reach the level of significance in MSS tumors (Figure 1E), suggesting that highly immunogenic MSI tumors may benefit more than MSS tumors from B cell-mediated antigen presentation for T-cell activation or antibody-dependent cellular cytotoxicity [44,45,46].

In the comprehensive analyses of in situ immune infiltrates Galon et al. noted close correlation between B cells (CD20+) and the T cell subset network within the CT and particularly with memory T cells at the IM region [47]. Later investigation of CRC intrametastatic immune infiltrates revealed the Immunoscore^®^ and TB score (Immunoscore^®^-like score combining CD8+ and CD20+ cells) to be the only parameters significantly associated with prolonged mCRC patient survival in multiple analysis [48]. Along with increased cytotoxic T cell and macrophage densities, elevated B cell counts were encountered in MSI tumors [27]. In addition, patient clusters with both high memory and cytotoxic T and B cell densities had prolonged disease-specific survival regardless of MSI status [27]. Our study therefore supports the added value of combined T and B cell prognostic power: the CD8+ and CD20+ Immunogradient indicators provided independent prognostic input in the multiple Cox regression models, importantly, pertinent to both MSS and MSI-CRCs. CD8-CD20 Imunogradient score provided significant prognostic stratifications both in MSS and MSI tumors (Figure 3B,C).

The infiltrative tumor growth pattern assessed by pathologist served as another independent feature and increased the prognostic power of the model. In agreement with the previous observations [49,50,51], IIS revealed the infiltrative tumor margin to be an adverse prognostic factor; IIS, score 0 identified the worst survival subgroup both in MSS and MSI tumors (Figure 3E,F). Inverse correlation between infiltrative tumor growth and the presence of immune response at the advancing tumor margin has been reported previously [52,53]. Although these associations may be of value for further TME studies, our data show that the tumor growth pattern maintains an independent prognostic value and indicates the need for robust assessment of this feature, similar to the efforts to quantify the tumor budding phenomenon in CRC [31,54]. Of note, tumor budding assessment by pathologist did not provide a prognostic value in our study.

Zlobec et al. reported a combined assessment of CD8+ cell infiltrates in TMA, tumor margin configuration data and lymph node spread to predict local recurrence in MSS CRCs; however, their study did not include MSI tumors that are commonly defined as non-budding tumors [55]. Another study introduced semi-quantitative Bayreuth score, based on tumor gland formation, budding and TIL analyzed in hematoxylin and eosin-stained whole slide images (WSI) to provide independent prognostic value along with TNM-staging for low-grade CRC [56]. Nearchou et al. proposed a spatial immuno-oncology index based on TIL and macrophages proximity analysis to tumor buds to provide highly significant combinatorial risk model for stage II CRC patient stratification [31,32]. Similarly, our study combines the prognostic power of tumor histology features and interacting immune cell subsets, yet with different spatial analysis methodologies applied. We show independent prognostic value for CD8+ and CD20+ cell infiltrates, measured by Immunogradient methodology, and the added prognostic value of the tumor growth pattern as IIS—an integrated prognostic biomarker for MSS and MSI CRCs.

The findings in the current study were achieved in a relatively small sample size cohort both for MSS and MSI-CRCs; larger cohort studies are needed to elucidate the role for immune cell populations and measurement methodologies for robust prognostic modeling. In addition, the prognostic power of the models achieved in this study remains to be directly compared to the Immunoscore^®^, immuno-oncology index and other systems proposed for practical implementation, which is best achieved in appropriately designed studies. Of note, the study was undertaken in patients who had not received neoadjuvant therapy. Tumor necrosis induced by neoadjuvant therapy is likely to change the tumor stromal community and thus the Immunogradient indicators. However, the nature of the change and its prognostic impact are currently unknown. Finally, tumor growth pattern reveals an independent prognostic value and therefore indicates the need for further efforts in tumor histology feature extraction to quantify growth pattern and budding.

## 4. Materials and Methods

### 4.1. Patients

A series of 99 cases of CRC which had undergone curative resection was retrieved from the archives of the Pathology Department at the Nottingham University Hospitals NHS Trusts. The series was selected to include 50 CRCs with MSI and 49 CRCs which were MSS and had been used to develop a screening test for Lynch Syndrome [57]. These cases had been tested by IHC for mismatch repair protein (MMR) for the purpose of either making a decision on adjuvant chemotherapy or for screening for Lynch Syndrome. Overall survival (OS) was defined as the time interval between first surgery and death due to any cause. Tumor samples of patients with preoperative treatment (n = 2), un-resected metastasis (n = 3), cases of mucinous cancer (i.e. >50% of the tumor section showing mucinous histology, n = 4) since a specific tissue classifier is needed, appendiceal tumor (n = 1), and tumor section area below <4.5 mm^2^ (n = 2) were excluded from further analyses. All tumor specimens were tested by IHC for expression of DNA MMR proteins, i.e., MLH1, PMS2, MSH2, MSH6, and by PCR followed by high-resolution melting analysis for MSI status and *BRAF*, *KRAS*, *PIK3CA* gene mutations, as described previously [57,58]. The *MLH1* gene promoter methylation analysis revealed MSI tumors to be mainly sporadic [57].Clinicopathological parameters and follow-up data of the final CRC patient cohort were obtained from the pathology reports and the clinical records and are summarized in Table 1.

### 4.2. Ethics Statement

Study approval and access for anonymized use of tumor tissue were granted by Nottingham Health Sciences Biobank (REC reference: 15/NW/0685).

### 4.3. Digital Image Acquisition and Analysis

IHC was performed on FFPE as previously described [57]. Four micrometer thick tissue sections were cut and stained for cytotoxic T cell marker CD8 (clone SP57, Roche Diagnostics, Mannheim, Germany), B cell marker CD20 (clone L26, Roche Diagnostics) and macrophage marker CD68 (clone KP1, Dako, Glostrup, Denmark). Slides were scanned at x20 objective magnification (0.5 μm resolution) using an Aperio AT2 Slide Scanner (Leica Microsystems, Wetzlar, Germany). Initial image analysis to segment tissue compartments was performed on WSIs using HALO™ software (version 2.2.1870; Indica Labs, Corrales, NM, USA). The tissue classification algorithm utilizes an artificial intelligence-based classifier trained to segment tissue into tumor, stroma, lymphoid follicles and background (i.e., necrotic areas, mucin pools, artifacts, and glass). The HALO Multiplex IHC algorithm (Version 1.2) was used to detect and extract coordinates of CD8+, CD20+, CD68+ cells.

### 4.4. Extraction of Interface-Zone and Immunogradient Indicators

The systematic extraction of the Immunogradient indicators was previously described [33]. In brief, the WSI of a tumor is processed by DIA software to identify tissue classes for each pixel and to extract coordinates and counts of positive cells. The DIA data is then subsampled by a hexagonal grid, as described in previous studies [59,60] and biomarker densities are calculated in each hexagon. The tumor edge (TE), which consists of hexagons on the interface between tumor and stroma, is computed based on changes in tissue class area fractions inside each hexagon; in Figure 2 the extracted TE are all yellow hexagons. The remaining hexagons are classified as either tumor, stroma or background, also by area fractions. Subsequently, the distance from each hexagon to the nearest TE is calculated. Using this distance, hexagons are ranked so that hexagons at the TE have rank 0 (distance 0), tumor-epithelium hexagons are assigned a rank equal to their distance from the nearest TE, while hexagons on the stromal side of the TE are assigned a rank equal to their *negative* distance to the nearest TE. This allows easy extraction of a tumor-stroma interface zone (IZ) of any width, e.g. an interface zone of width 9 would cover ranks [−4; 4]. For CRC, an IZ of width 3 (ranks [−1; 1]), abbreviated IZ_3_, was previously found optimal [33]. The tumor aspect (T) (rank = 1) and stroma aspect (S) (rank = −1) of IZ_3_ are highlighted in Figure 2 as the red and green hexagons, respectively. From the extracted IZ, simple Immunogradient indicators can be calculated, for example, mean CD8+ cell densities in the tumor aspect and in the stroma aspect. Additionally, the ranking allows plotting biomarkers gradient profiles across the interface zone (Figure 2A); and computing indicators like the Center of mass (CM) which estimates which part the cell density gravitates towards:

Center of mass (CM):

$$\mathrm{CM}(q)\;=\;\frac{\sum_{r_{i}}r_{i}\;q\left(r_{i}\right)}{\sum_{r_{i}}q\left(r_{i}\right)}$$

where r_i_ indexes the IZ_3_ ranks, r_i_ ∈ [−1;1], and q(r_i_) denotes the rank statistics, e.g., the mean of CD8+ cell density.

In addition to the Immunogradient indicators, the intratumoral CD8+, CD20+ and CD68+ cell densities, i.e., densities in all tumor tissue, were extracted.

### 4.5. Statistic Analyses

Fisher’s exact test was used to examine the associations between clinicopathological parameters and MSI status. Since immune cell density distributions in the CRC samples showed left asymmetry (by the Kolmogorov–Smirnov test), cell density indicators were log-transformed for parametric statistics. The statistical significance of cell density variations in the aspects of IZ_3_ and tumor compartment were tested by one-way ANOVA followed by Bonferroni’s post-hoc test for pairwise comparisons and a two-sided Welch’s *t*-test for homogeneity of variances. Cutoff Finder [38] was used to obtain an optimal cut-off value for cell density indicators to test their interrelationships and predictions of OS. Univariate and multivariate survival analyses were performed using Cox proportional hazards models obtained by a stepwise likelihood ratio test. A leave-one-out cross-validation was used to analyze the relevance of the selected indicators, to compare the accuracy of predictive models [61]. All statistical tests were two-sided and conducted at a nominal significance level of 0.05. Statistical calculations were performed by SAS software (version 9.4; SAS Institute Inc., Cary, NC, USA); graphs were generated by R (version 4.0.0; R Foundation for Statistical Computing, Vienna, Austria) and GIMP (version 2.10.14; The GIMP team, www.gimp.org).

## 5. Conclusions

In summary, we present a novel combinatorial prognostic model for MSS and MSI CRC patients, based on 3 independent features: IZ Immunogradient indicators of CD8+ and CD20+ cell densities and infiltrative tumor growth pattern assessed by pathologist. The immuno-interface score, IIS, was superior to TNM-staging and molecular features, and displayed as strong predictor of patient outcomes in both MSS and MSI tumors.

## Figures and Tables

**Table 1:** Clinicopathological parameters in patients grouped by tumor microsatellite instability status.

Clinicopathological Parameters		MSS CRC, *n* (%)	MSI CRC, *n* (%)	*p-*Value *
	Total	48 (100)	39 (100)	-
OS follow-up, months	Median	52	46	-
	Range	2–97	1–117	
Deceased	5-year follow-up	11 (12.6)	17 (19.5)	-
	10-year follow-up	11 (12.6)	18 (20.7)	
Age groups by median	≤71 years	32 (66.7)	13 (33.3)	0.0026 *
	>71 years	16 (33.3)	26 (66.7)	
Sex	Female	23 (47.9)	26 (66.7)	0.0878
	Male	25 (52.1)	13 (33.3)	
TNM stage	I	0	1 (2.6)	0.9999
	II	31 (64.5)	23 (58.9)	
	III	16 (33.3)	13 (33.3)	
	IV	1 (2.1)	2 (5.1)	
Histological grade (G)	G2	44 (91.7)	20 (51.3)	<0.0001 *
	G3	4 (8.3)	19 (48.72)	
Tumor invasion (pT)	pT2	1 (2.1)	1 (2.6)	0.8115
	pT3	36 (75)	27 (69.2)	
	pT4	11 (22.9)	11 (28.2)	
Lymph node metastasis (pN)	pN0	32 (66.7)	25 (64.1)	0.9027
	pN1	8 (16.7)	8 (20.5)	
	pN2	8 (16.7)	6 (15.4)	
Distant metastasis (M)	M0	47 (97.9)	37 (94.9)	0.5850
	M1	1 (2.1)	2 (5.1)	
Lymphovascular invasion (LVI)	LVI0	28 (58.3)	24 (61.5)	0.8279
	LVI1	20 (41.7)	15 (38.5)	
Perineural invasion (Pne)	Pne0	42 (87.5)	32 (82.1)	0.5529
	Pne1	6 (12.5)	7 (18.9)	
Tumor location	Left	28 (58.3)	3 (7.7)	<0.0001 *
	Transverse	0	1 (2.56)	
	Right	19 (39.6)	33 (84.6)	
	Multiple sites	1 (2.1)	2 (5.1)	
Tumor growth pattern	Pushing margin	23 (47.9)	26 (66.7)	0.0878
	Infiltrative margin	25 (52.1)	13 (33.3)	
Tumor budding	Low	33 (68.8)	25 (64.1)	0.6557
	High	15 (31.2)	14 (35.9)	
Peritumoral lymphocytes	Inconspicuous	35 (72.9)	20 (52.6)	0.0707
	Conspicuous	13 (27.1)	18 (47.4)	
*BRAF* mutation status	Wild-type	44 (91.7)	18 (46.2)	<0.0001 *
	Mutant	4 (8.3)	21 (53.8)	
*KRAS* mutation status	Wild-type	25 (52.1)	32 (82.2)	0.0060 *
	Mutant	23 (47.9)	7 (17.9)	
*PIK3CA* mutation status	Wild-type	40 (83.3)	31 (79.5)	0.7822
	Mutant	8 (16.7)	8 (20.5)	

* *p-*value < 0.05 is considered significant.

**Table 2:** Summary statistics of the Immunogradient and intratumoral immune cell density indicators in patients grouped by tumor microsatellite instability status.

Immunogradient and Intratumoral Cell Density (Cells/mm^2^) Indicators	MSS CRC, n = 48			MSI CRC, n = 39			
	Mean	Median	sd	Mean	Median	sd	*p-*Value *
CD8_CM	−0.35	−0.35	0.17	−0.20	−0.18	0.21	0.0006 *
CD8_d_S	193.78	147.06	147.73	370.76	294.91	404.69	0.0024 *
CD8_d_TE	141.82	90.03	128.49	339.94	208.15	400.52	0.0004 *
CD8_d_T	76.47	49.24	92.49	262.40	140.22	342.64	0.0001 *
INT_CD8	65.37	37.59	81.99	238.90	133.46	311.26	<0.0001 *
CD20_CM	−0.49	−0.54	0.23	−0.59	−0.63	0.14	0.0141 *
CD20_d_S	54.26	32.81	68.44	71.37	36.78	83.35	0.3650
CD20_d_TE	31.61	14.01	59.39	30.56	18.93	33.44	0.7857
CD20_d_T	12.20	4.66	30.68	5.40	3.87	6.12	0.0899
INT_CD20	13.75	4.19	31.21	9.70	5.88	12.90	0.6003
CD68_CM	−0.26	−0.28	0.14	−0.11	−0.08	0.14	<0.0001 *
CD68_d_S	173.95	158.15	118.19	182.45	173.88	104.31	0.5616
CD68_d_TE	145.14	120.25	99.73	190.06	175.17	106.06	0.0281 *
CD68_d_T	72.49	55.29	73.41	126.52	100.39	82.40	<0.0001 *
INT_CD68	60.04	48.90	55.89	112.15	95.33	71.48	<0.0001 *

CD8_d, CD20_d and CD68_d indicate mean density for each biomarker, respectively; densities are summarized over hexagons in the stroma aspect (S), tumor edge (TE) and tumor aspect (T) of the IZ; INT—mean intratumoral density; CM—Center of Mass implicitly by mean cell density in ranks; sd—standard deviation of indicator. * *p-*value < 0.05 is considered significant.

**Table 3:** Statistics of univariate analyses of clinicopathological parameters, Immunogradient and intratumoral immune cell density indicators for patient overall survival.

Clinicopathological Parameters, Immunogradient and Intratumoral Cell Density Indicators	CRC, n = 87		
	HR	95% CI	*p*-Value *
Age group (>median vs. ≤median)	1.33	0.64–2.77	0.4480
Sex (male vs. female)	0.84	0.40–1.77	0.6481
TNM stage (III-IV vs. I-II)	1.06	0.49–2.30	0.8825
pT status (pT4 vs. pT2-3)	1.05	0.45–2.46	0.9151
pN status (pN1-2 vs. pN0)	0.98	0.45–2.18	0.9683
M status (M1 vs. M0)	3.41	0.80–14.60	0.0978
G stage (G3 vs. G2)	1.60	0.74–3.46	0.2312
LVI status (LVI1 vs. LVI0)	1.77	0.56–2.43	0.6737
Pne status (Pne1 vs. Pne0)	1.67	0.68–4.12	0.2648
Tumor location (right/transverse/multiple vs. left)	2.00	0.85–4.68	0.1128
Tumor growth pattern (infiltrative vs. pushing margin)	2.81	1.32–5.98	0.0075 *
Tumor budding (high vs. low)	2.05	0.98–4.29	0.0556
Peritumoral lymphocytes (inconspicuous vs. conspicuous)	1.28	0.61–2.69	0.5234
MSI status (MSI vs. MSS)	2.07	0.97–4.43	0.0614
*BRAF* status (mutant vs. wild-type)	0.98	0.44–2.18	0.9501
*KRAS* status (mutant vs. wild-type)	0.78	0.36–1.72	0.5369
*PIK3CA* status (mutant vs. wild-type)	0.59	0.21–1.70	0.3264
CD8_CM (high vs. low)	0.31	0.15–0.66	0.0013 *
CD8_d_S (high vs. low)	1.46	0.64–3.31	0.3600
CD8_d_TE (high vs. low)	0.64	0.31–1.35	0.2400
CD8_d_T (high vs. low)	0.53	0.25–1.10	0.0850
INT_CD8 (high vs. low)	2.13	0.93–4.88	0.0670
CD20_CM (high vs. low)	0.39	0.16–0.91	0.0230 *
CD20_d_S (high vs. low)	0.30	0.12–0.75	0.0061 *
CD20_d_TE (high vs. low)	0.33	0.10–1.08	0.0530
CD20_d_T (high vs. low)	0.43	0.20–0.90	0.0210 *
INT_CD20 (high vs. low)	0.41	0.18–0.90	0.0230 *
CD68_CM (high vs. low)	1.77	0.84–3.74	0.1300
CD68_d_S (high vs. low)	0.59	0.28–1.23	0.1500
CD68_d_TE (high vs. low)	0.65	0.30–1.43	0.2800
CD68_d_T (high vs. low)	1.82	0.77–4.26	0.1600
INT_CD68 (high vs. low)	1.73	0.79–3.81	0.1700

HR hazard ratio, CI confidence interval. * *p*-value < 0.05 is considered significant.

**Table 4:** Multiple Cox regression models for patient overall survival.

Clinicopathological and Immunogradient Indicators	CRC, n = 87		
**Model#1, LR: 23.03; *p* < 0.0001**	**HR**	**95% CI**	***p-*** **Value**
CD8_CM (high)	0.31	1.42–0.67	0.0029
CD20_CM (high)	0.33	0.14–0.78	0.0113
Tumor growth pattern (infiltrative)	2.90	1.34–6.29	0.0071
**Model#2, LR: 15.50; *p* = 0.0004**	**HR**	**95% CI**	***p-*** **Value**
CD8_CM (high)	0.30	0.14–0.64	0.0019
CD20_CM (high)	0.37	0.16–0.87	0.0228

HR hazard ratio, CI confidence interval, LR likelihood ratio.

## Supplementary Materials

The following are available online at https://www.mdpi.com/2072-6694/12/10/2902/s1, Figure S1: An image of original CD8 IHC tissue section which was used for IZ overlay in Figure 2.
