# Supplementary material for: Immuno-Interface Score to Predict Outcome in Colorectal Cancer Independent of Microsatellite Instability Status
Source: Cancers (Basel). 2020 Oct 9;12(10):2902. doi: 10.3390/cancers12102902 (PMC7600992; doi:10.3390/cancers12102902)
Supplement: Supplementary file 1 [file cancers-12-02902-s001.pdf]

cancers

Article

Supplementary Material: Immuno-Interface Score to Predict Outcome in Colorectal Cancer Independent of Microsatellite Instability Status

Ausrine Nestarenkaite [1,2,*], Wakkas Fadhil [3,4], Allan Rasmusson [1,5], Susanti Susanti [3,4], Efthymios Hadjimichael [3,4], Aida Laurinaviciene [1,5], Mohammad Ilyas [3,4] and Arvydas Laurinavicius [1,5]

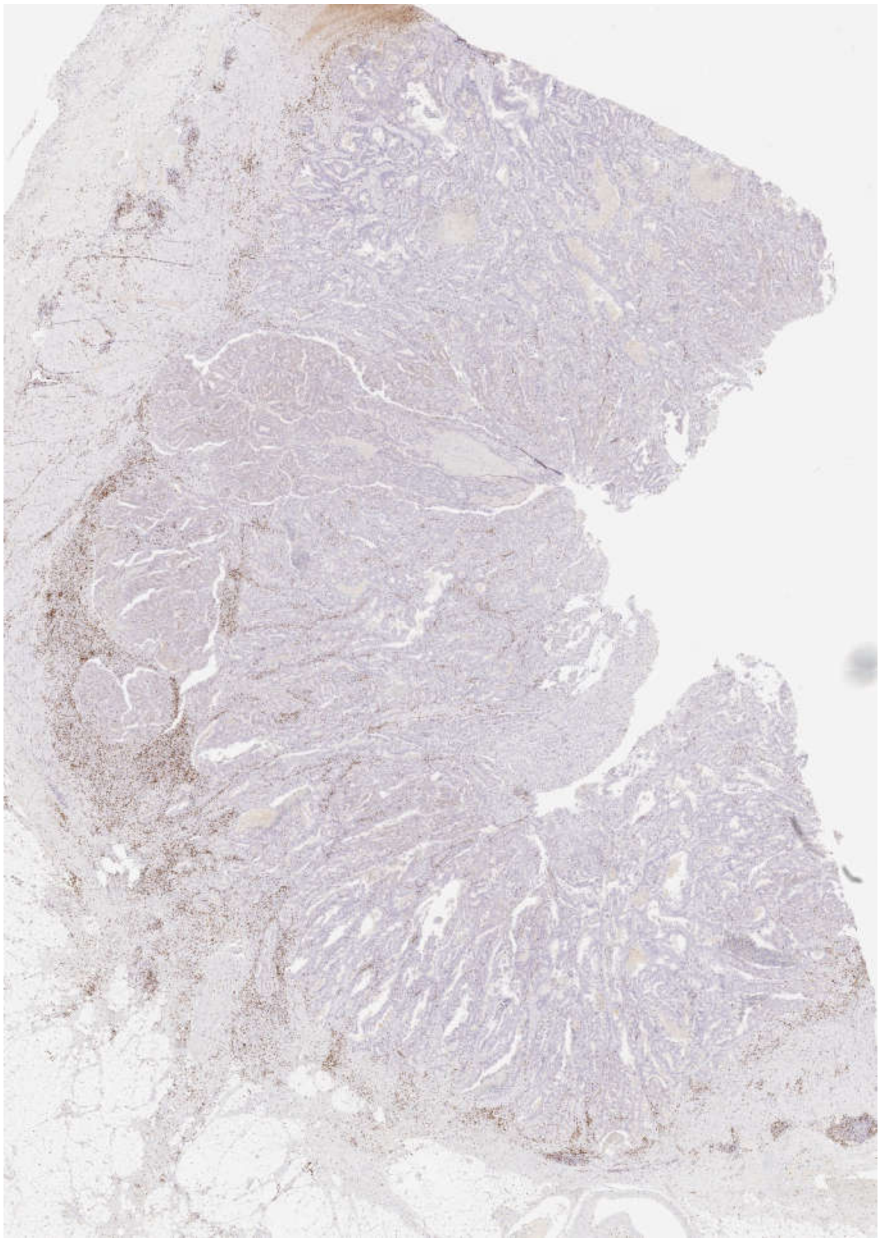

Figure S1. An image of original CD8 IHC tissue section which was used for IZ overlay in Figure 2.

© 2020 by the authors. Submitted for possible open access publication under the terms and conditions of the Creative Commons Attribution (CC BY) license (http://creativecommons.org/licenses/by/4.0/).
